# Carrier Solvents of Electronic Nicotine Delivery Systems Alter Pulmonary Surfactant

**DOI:** 10.1021/acs.chemrestox.0c00528

**Published:** 2021-05-04

**Authors:** Nathalie Hayeck, Carl Zoghzoghi, Ebrahim Karam, Rola Salman, Nareg Karaoghlanian, Alan Shihadeh, Thomas Eissenberg, Salah Zein El Dine, Najat A. Saliba

**Affiliations:** † Chemistry Department, Faculty of Arts and Sciences, American University of Beirut, Beirut 1107-2020, Lebanon; ‡ Center for the Study of Tobacco Products, Department of Psychology, Virginia Commonwealth University, Richmond, Virginia 23284, United States; § Mechanical Engineering Department, Maroun Semaan Faculty of Engineering and Architecture, American University of Beirut, 1107-2020 Beirut, Lebanon; ∥ Department of Psychology, Virginia Commonwealth University, Richmond, Virginia 23284, United States; ⊥ Department of Internal Medicine, Faculty of Medicine, American University of Beirut, 1107-2020 Beirut, Lebanon

## Abstract

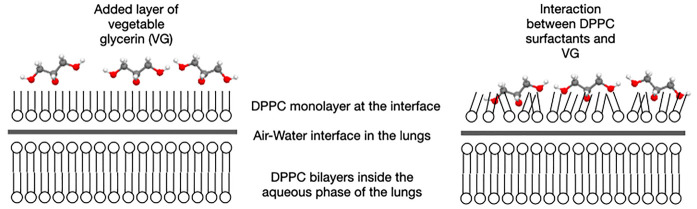

In late 2019, hundreds of users of electronic products that aerosolize a liquid for inhalation were hospitalized with a variety of respiratory and gastrointestinal symptoms. While some investigations have attributed the disease to the presence of vitamin E acetate in liquids that also contained tetrahydrocannabinol, some evidence suggests that chronic inhalation of two common solvents used in electronic nicotine delivery systems (ENDS), propylene glycol (PG) and vegetable glycerin (VG), can interfere with the lipid components of pulmonary surfactant and cause or exacerbate pulmonary injury. The interaction between PG, VG, and lung surfactant is not yet understood. This study presents an examination of the molecular interactions of PG and VG with lung surfactant mimicked by 1,2-dipalmitoyl-*sn*-glycero-3-phosphocholine (DPPC). The interaction of DPPC and PG-VG is studied by attenuated total reflectance fourier transform infrared spectroscopy. The results showed that PG and VG altered the molecular alignment of the DPPC surfactant. The orientation of the surfactant at the surface of the lung affects the surface tension at the air–water interface, thereby influencing breathing. These findings suggest that chronic aerosolization of the primary solvents in ENDS might alter the function of pulmonary surfactant.

## Introduction

Electronic nicotine delivery systems (ENDSs) have gained popularity especially among teenagers and young adults due to their effectiveness in delivering the stimulant drug nicotine in thousands of appealing flavors.^1,2^ ENDS-associated lung injury and/or diseases such as acute respiratory distress syndrome (ARDS) and certain types of pneumonia have been reported since 2012.^3−8^ In 2019, hundreds of cases of electronic cigarette or vaping product use-associated lung injury (EVALI) were reported by the United States (US) Centers for Disease Control and Prevention (CDC). Since then, 2807 hospitalized EVALI cases and 68 deaths have been confirmed in the US and some cases have been found in Argentina, Canada, Great Britain, and Malaysia.^9^ Vitamin E acetate, an additive in some cannabinoid-containing vaping products, was identified as one cause of EVALI because it was found in lung fluid samples from EVALI patients.^10^ However, 29% of fatal cases and 14% of nonfatal cases have reported exclusive use of ENDS products.^11^ A recent study has shown that a physical interaction between vitamin E acetate and pulmonary surfactant reduces the elastic properties of the surfactant, inducing their failure.^12^ Nevertheless, the authors indicated that more evidence is needed to confirm the effect of vitamin E acetate and to identify whether other factors play a role in EVALI.

There is evidence that ENDS product use can cause pulmonary injury/disease. For example, a recent longitudinal study using the data files from the Population Assessment of Tobacco and Health (PATH) of 32,000 American adults has confirmed that exclusive ENDS use is a risk factor for respiratory disease, while the dual use of ENDS and combustible cigarettes increases the risk compared to using one of the two products alone.^13^ Also, chronic exposure to aerosols from nonflavored nicotine-containing liquids alters the inflammation profile of the lungs and increases the risk of acute lung injury (ALI).^14^ Also, long-term exposure to common ENDS liquid ingredients, propylene glycol (PG) and vegetable glycerin (VG), without nicotine might affect airway resistance,^15^ alter pulmonary surfactant homeostasis and influence alveolar macrophages, increase phospholipids in the airways,^16^ and increase inflammation.^17,18^

The exact mechanism by which ENDS causes lung injuries is still unknown. One of the proposed hypotheses suggests a two-step action consisting of PG-VG aerosols affecting the homeostatic state of the pulmonary immune cells followed by the inhalation of other chemicals that trigger inflammation.^19^ In fact, surfactant, which is present at the interface between the alveolar fluid and the air space, is the first barrier of the pulmonary tissues. Its role is to prevent the collapsing of the alveoli during breathing by reducing its surface tension.^20^ Surfactant dysfunction is characteristic of patients with ALI and ARDS.^21,22^ Pulmonary surfactant is constituted of lipids (90%) and proteins (10%), where 80% of the lipid fraction is phosphatidylcholines. The major component of these phosphatidylcholines is phospholipid 1,2-dipalmitoyl-*sn*-glycero-3-phosphocholine (DPPC), representing around 40%.^23^

This study uses an *in vitro* model to investigate the effects of the interaction between the primary constituents of all ENDS liquids (PG-VG) and the pulmonary surfactant mimicked by the dominant molecule (DPPC). The molecular interactions between the two systems are examined using attenuated total reflectance-fourier transform infrared (ATR-FTIR) spectroscopy.

## Materials and Methods

### Materials

The surfactant DPPC (Avantis Polar Lipids, USA; lot: 850355P-1G-A-3232) was of analytical grade and used as received without any further purification. Chloroform (CHCl_3_) of analytical grade (≥99.9%) was used as the spreading solvent for DPPC, PG (Sigma-Aldrich, 99.5%), and VG (Sigma-Aldrich, 99–101%) solutions. CHCl_3_ and ethanol were used to clean the surface of the ATR cell. A SMOK brand atomizer (V8–T8; 0.15 Ω; 50–260 W) powered by a 3 Li–Po battery pack (900 mAh–11.1 V) and connected to a LAVABOX DNA board was used to heat pure PG or VG liquids separately. A zero air cylinder (purity >99.99%) was used as a gas supply for the ENDS device. FTIR spectra were recorded using a Nicolet Avatar 360 Fourier Transform equipped with a multireflection horizontal ATR flow-through cell (REFLEX analytical corporation) permitting 10 reflections on a ZnSe surface of 5.11 cm^2^. Spectra between 400 and 4000 cm^–1^ were collected at a resolution 1 cm^–1^ (0.48 cm^–1^ data spacing) and background corrected.

### Methods

#### Experimental Setup

**Figure 1:**
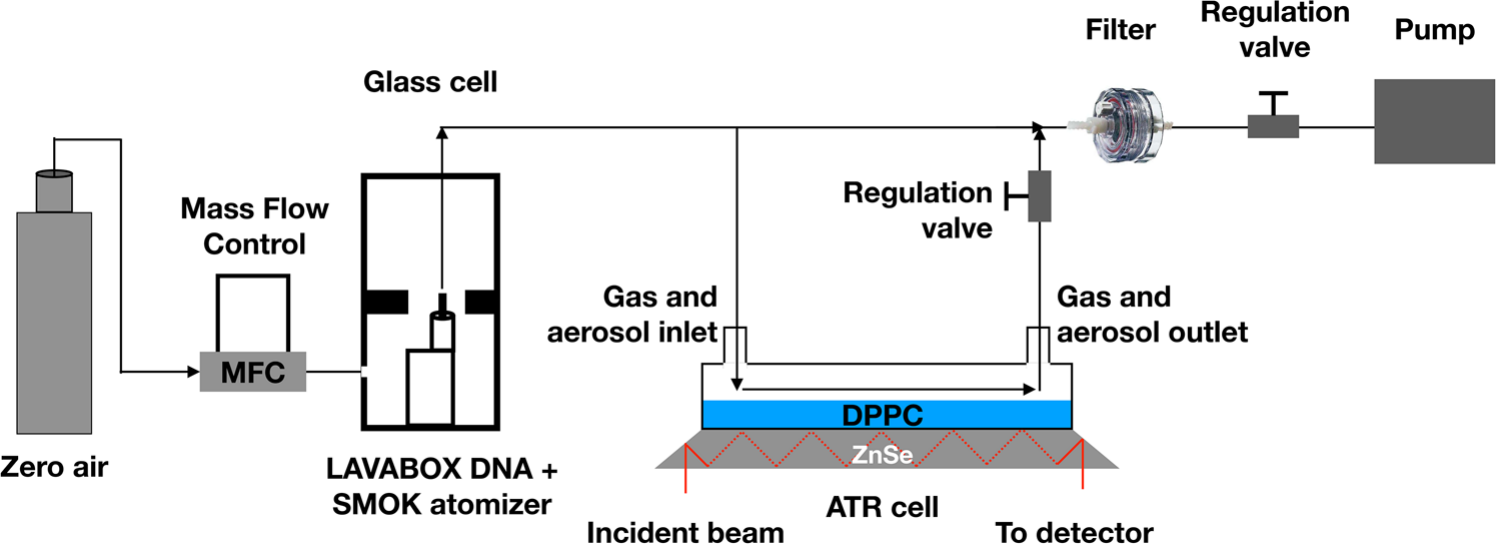
Experimental setup used to generate vaping aerosols by the ENDS device and their flow through the ATR-FTIR cell.

Figure 1 illustrates the experimental setup used to generate puffs and to deposit the generated aerosols on the ATR-FTIR cell. The ENDS device was operated at 70W, well below its rated 260W limit, to minimize formation of PG-VG degradation products.^24^ The puffing parameters consisted of 1 L/min flow, 4 s puff duration, and 10 s interpuff interval. The device was programmed and controlled using the Escribe software from Evolv (Escribe2_SP17_INT) as well as through serial commands sent from a LabVIEW program. The optimized 70 mL/min of the total flow was oriented toward the gas/ATR cell to allow PG or VG aerosols to interact with the deposited DPPC in a manner similar to the deposition of aerosols onto the pulmonary surfactant. Pure PG or VG liquids were vaped separately to assess the effect of each one of these humectants. The ENDS device was placed in a glass chamber and exposed to an excess of zero air flow to maintain a low level of humidity and CO_2_ which can otherwise saturate the FTIR spectra. A vacuum pump was used to draw the zero air into the ENDS device during the puffing process to eliminate the contamination from any external source. The ENDS device airflow inlet slits were set to the maximum open position. The flow rate entering the ENDS mouthpiece was controlled during each session to ensure that the air entering the ENDS device was maintained constant at the set flow rate of 1L/min.

The blank spectrum was obtained after cleaning the ATR surface with 200 μL of chloroform and left to completely evaporate. Ten consecutive puffs constituted one vaping session, and seven sessions of 10 cumulative puffs each (70 puffs total) were completed. FTIR spectra of 256 scans each were collected in triplicate after each session. The set of seven sessions was repeated three times. Unless stated otherwise, all the FTIR spectra were normalized by the blank spectrum before analysis.

#### ATR Gas Cell and FTIR Analysis

To model the surfactant layer at the alveoli–air interface, 200 μL of a solution containing 1 mg/mL of the pulmonary surfactant, DPPC, in chloroform was spread on an attenuated total reflectance (ATR) surface of ZnSe. The ATR is cleaned using chloroform then ethanol solvents. The interaction of DPPC with PG and VG was first tested by depositing 200 μL of PG or VG solutions which were left to sit 10 min for solvent evaporation before FTIR scanning. Initial solutions of PG or VG were prepared in chloroform/ethanol (90/10 v/v) solvents at a concentration of 100 mg/mL. These solutions were diluted in chloroform to prepare final solutions at a concentration of 10 mg/mL. This high concentration was chosen so that the effects of PG or VG on the adsorbed DPPC are enhanced and easily detectable with FTIR.

The adsorption of gases and aerosols was tested using a gas cell mounted on top of the ATR crystal. The mixture of gases and aerosols emitted from the electronic cigarette was directed to the gas cell where the DPPC was deposited onto the ATR crystal (Figure 1). Before each FTIR scan, 10 puffs of pure VG or PG were passed through the gas cell to allow for sufficient interaction.

## Results and Discussion

### Results

**Figure 2:**
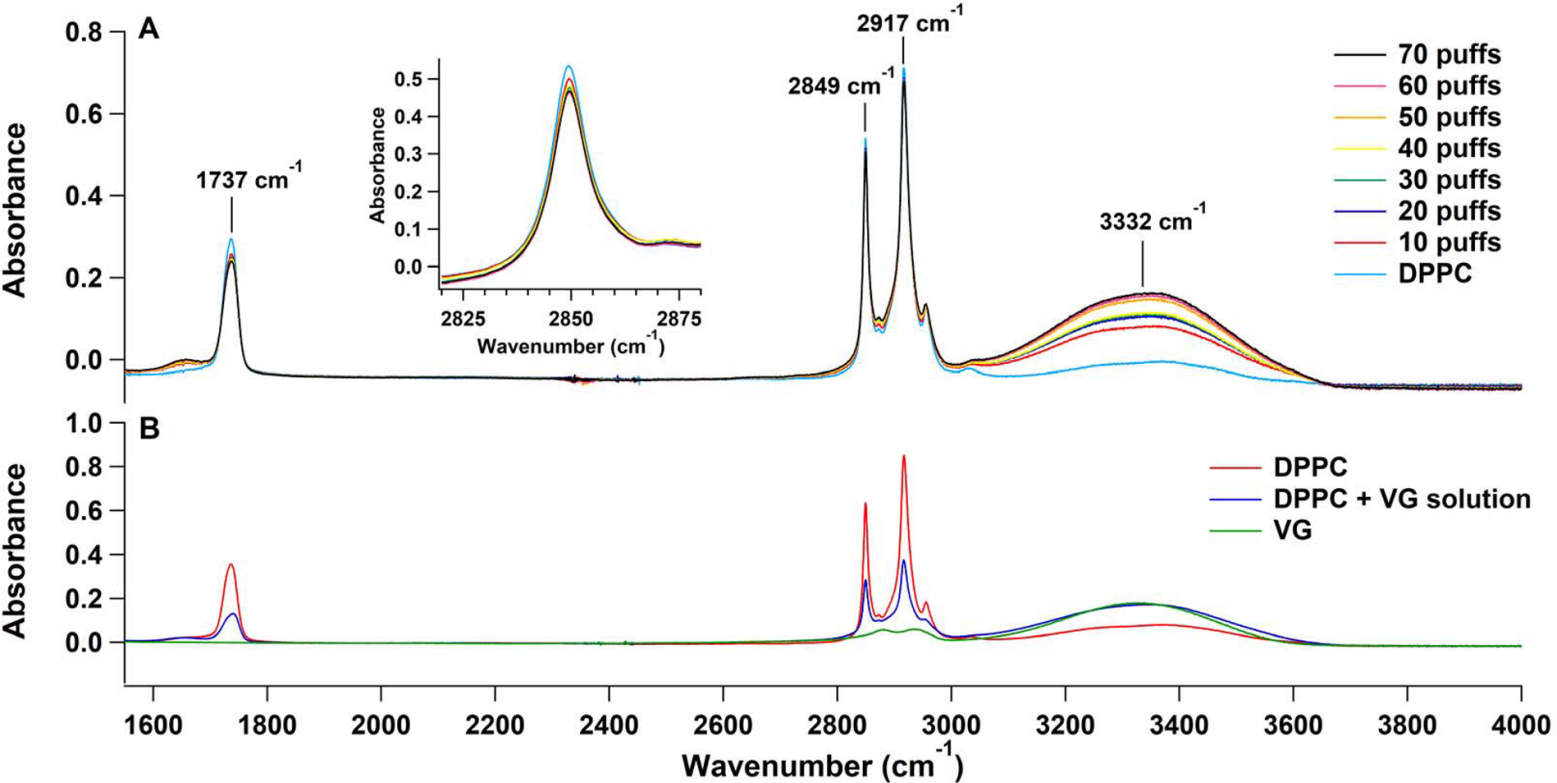
(A) FTIR spectra of DPPC and after each session of 10 puffs of VG using the ENDS device. (B) ATR-FTIR spectrum of a concentrated VG solution placed on a clean ATR surface; a spectrum of DPPC placed on a clean ATR surface (in red), and the spectrum of the same DPPC after the addition of the concentrated VG solution (in blue).

Figure 2A shows a section (from 1500 to 4000 cm^–1^) of the FTIR spectrum of the DPPC molecules that were deposited on the ATR ZnSe crystal and the spectra of the 10–70 puffs of VG aerosols that were added sequentially afterward. The 3332 cm^–1^ peaks, which correspond to the stretching frequency of the OH groups in VG, increased with the increase of the number of puffs, reflecting the greater amounts of adsorbed VG onto the DPPC-ATR surface. Simultaneously, the DPPC related vibrational bands at 1737 cm^–1^ (carbonyl, C=O peak) and 2849 and 2917 cm^–1^ (methylene CH_2_ symmetric and antisymmetric stretching peaks, respectively) decreased when the number of puffs increased (Figure 2A). A concentrated VG liquid solution prepared in chloroform was placed on top of the DPPC. As shown in Figure 2B, the spectra in green and red correspond to a concentrated solution of VG and

DPPC solution deposited onto a clean ATR surface, respectively, whereas the blue spectrum reflects the added VG solution on top of the adsorbed DPPC layers. Similar to what was observed after adding the VG aerosols, adding the VG liquid solution to the DPPC layers caused a decrease in the 1737, 2849, and 2917 cm^–1^ vibrational bands.

**Figure 3:**
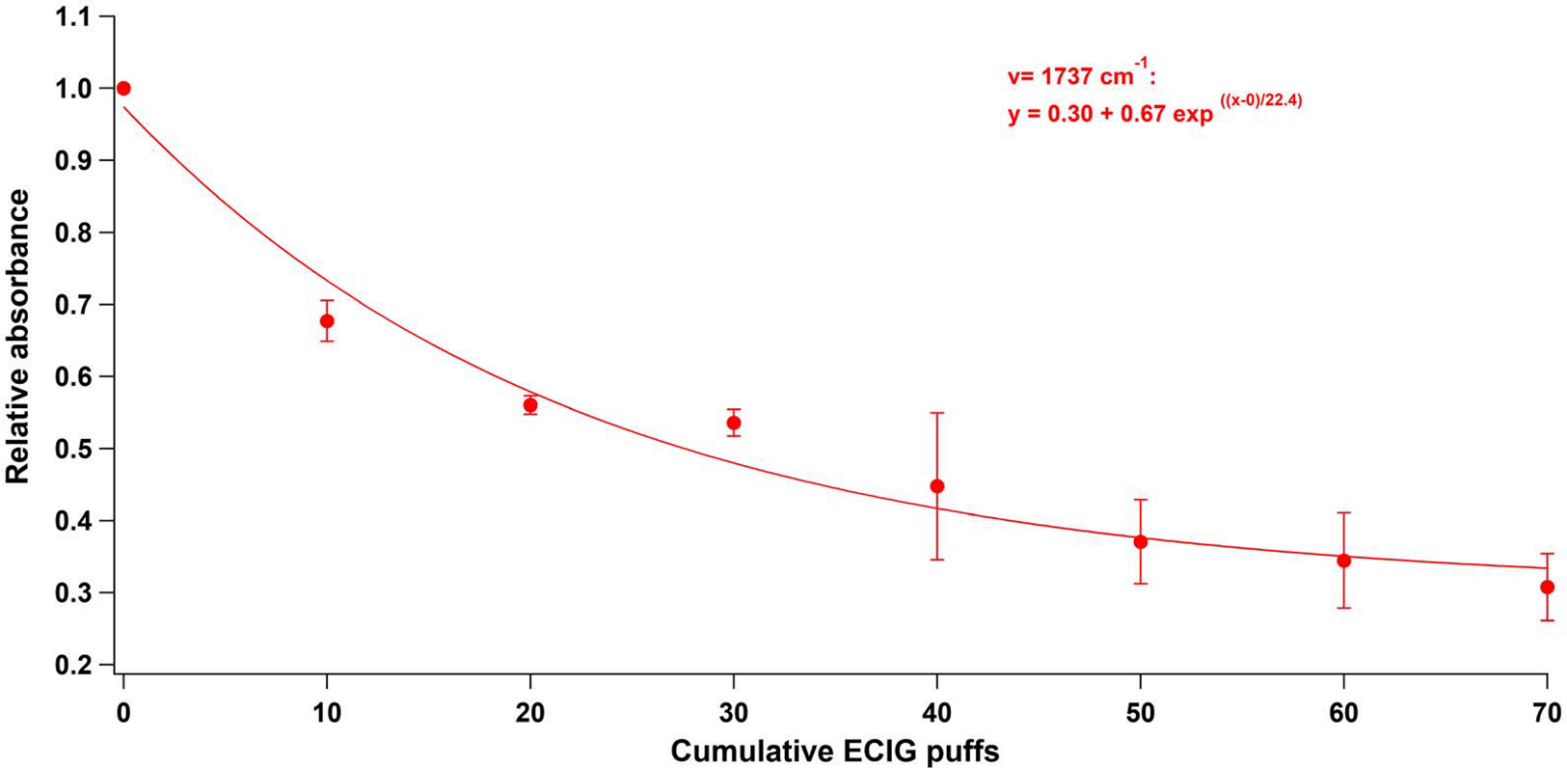
Relative abundance of one of the affected DPPC vibrational bands (1737 cm^–1^) as a function of the cumulative number of puffs.

The cumulative addition of VG puffs to the deposited DPPC caused the relative intensities of the specific peaks of DPPC (1737, 2849, and 2917 cm^–1^) to decay exponentially (Figure 3). Illustrated in Figure 3 is the relative absorbance of one of the specific vibrational bands of DPPC, that is, the carbonyl stretch (1737 cm^–1^) against the cumulative number of puffs. Note that the 1737 cm^–1^ IR peak was normalized by the first deposited DPPC materials and by the 3332 cm^–1^ OH peak of VG to account for the variability in the amount of aerosols deposited on DPPC. The error bars represent the standard deviation (±SD) obtained from three repetitions of each vaping session. Similar behaviors, although to a lesser extent, were also observed with PG liquid solutions and aerosols (Figure S3).

### Discussion

The decrease in the intensities of the DPPC peaks at 1737, 2849, and 2917 cm^–1^ could be due to three possible scenarios: (1) The effective IR light penetration thickness is weakened due to the added VG layers, (2) the consumption of DPPC via a chemical reaction, or (3) the change in the orientation of DPPC molecule toward the surface.^25^

Scenario (1) was ruled out by adding DPPC on top of the DPPC and VG mixture and observing an increase in the DPPC peaks without showing a decrease in VG peaks. This manipulation confirmed that the light penetration of the beam is effective through the different materials.^26^ Scenario (2) was also eliminated since no new FTIR peaks were found when a solution of VG was added to DPPC, which supports the notion that no new reactions between DPPC and VG took place. Hence, scenario (3) which posits an ability of VG to change the orientation of the DPPC adsorbed on the ATR crystal is the most plausible.

The ATR-FTIR technique is widely used to study the orientation and structural characterization of surfactant and other types of films.^27−30^ This technique is based on the fact that a maximal absorption of the IR light is obtained when the transition dipole moment of a molecular vibration is parallel to the electrical field component of the incident light and minimal when the two vectors are perpendicular.^28^ Consequently, we suggest that the decrease in the intensities observed is caused by the change in the orientation of the transition dipole moment of the vibration mode in the DPPC molecule.^25,30^ These results are in good accordance with changes in the ability of pulmonary surfactant to lower the surface tension observed upon the addition of a high concentration of ENDS aerosols.^31^

The role of surfactant in reducing the surface tension of the lung during respiration is suggested to be based on DPPC bilayer aggregates that are present in the lungs and able to diffuse to the air–liquid interface with the help of phospholipids and proteins to form a DPPC monolayer.^32^ On the other hand, the presence of glycerol (VG) in a water-DPPC solution was shown to be partitioned equally near the lipid membrane surface and in the bulk.^33^ One conclusion based on these two observations is that VG can interact with the DPPC bilayers in the aqueous solution of the lungs and the DPPC monolayer present at the interface. Even if our findings could not confirm the morphology of the DPPC layers on the ATR crystal, FTIR results showed that VG interacted with DPPC. The interaction between glycerol and phosphatidylcholine bilayers was reported to change the position of DPPC chains from tilted to interdigitated and perpendicular to the plane of the membrane.^34,35^ Another study also showed that glycerol increases the bending stiffness of the surfactant monolayer formed by DPPC/POPG (palmitoyloleoylphosphatidylglycerol).^36^ This result is explained by the fact that glycerol usually substitutes water molecules in solvation layers and concentrates in the interfacial region,^34^ creating a thin adlayer under the lipid film.^36^ This behavior affects the mechanical stability of pulmonary surfactant, leading to a depletion of surfactant from the pulmonary interface.

The size distribution of aerosols generated by ENDS systems is affected by several parameters (presence of nicotine and vanillin, increasing power, and percentage of PG and VG),^37,38^ which implies a variation of aerosols deposition in different regions of the lungs. Nevertheless, all the tested conditions lead to alveolar deposition, representing more than 25% of the deposited particles in the lungs.^38^ Besides, an estimated 175 puffs that are inhaled by one ENDS user per day would lead to a volume of aerosols per unit surface area around 5–9-fold higher than the volume per unit surface area of the surfactant layer.^39^ These two findings show the importance of the results shown in Figure 3 which represent the modification in the orientation of the DPPC molecules with the increasing number of puffs.

Once inactivated, DPPC can no longer protect the alveoli from contaminants^40,41^ which might originate from ENDS vaping or other environmental sources (air pollution) causing thereby inflammation, which might lead to some of the EVALI and lung diseases related to ENDS use.

This study suggests that a molecular interaction between the major phospholipid present in human lung, DPPC, and VG or PG aerosols leads to a change in the surface DPPC alignment. Extrapolations to comment on compressibility modulus and reduction of surface tension are still the subject of many discussions. A review of the literature showed in one of the studies that the effect of ENDS aerosol on pulmonary surfactant induces a shift in the compression isotherm of the surfactant film.^42^ The authors deduced that this impairment of the surfactant function might result in adverse effects on breathing. Recently, Sosnowski et al.^31^ controlled the amount of VG and PG deposited on the surfactant film and showed that these compounds can have physicochemical effects on pulmonary surfactant leading to altering the ability of the lungs to reduce their surface tension during respiration. However, Przybyla et al.^43^ showed that ENDS aerosol has no effect on the compression isotherm of the surfactant but can cause alterations in lateral arrangement of the surfactant at the interface. Discrepancies were due mainly to the different methods and varying concentrations of ENDS aerosol that were dosed onto the surfaces. Therefore, future studies might focus on measuring surface tension using the Langmuir balance to ensure the formation of a DPPC monolayer on the water surface under known concentrations of ENDS aerosols.

In summary, there is an important need to study the effect of PG and VG aerosols on pulmonary surfactant *in situ* to unravel the molecular level of interaction between the surfactant and ENDS carrier solvents, to understand how they are incorporated between the DPPC molecules, and to determine at what percentage of VG and PG the surfactant activity will be altered.

## Supporting Information Available

Figure S1: ATR-FTIR spectrum of DPPC between 750 and 3100 cm^–1^ with an indication of its specific vibrational absorbance. Figure S2: FTIR spectra of DPPC and after each session of 10 puffs of PG using the ENDS device. Figure S3: Relative abundance of one of the affected DPPC vibrational bands (1737 cm^–1^) as a function of the cumulative number of puffs of VG and PG (PDF)
