# Supplementary material for: Carrier Solvents of Electronic Nicotine Delivery Systems Alter Pulmonary Surfactant
Source: Chem Res Toxicol. 2021 May 4;34(6):1572–7. doi: 10.1021/acs.chemrestox.0c00528 (PMC8220501; doi:10.1021/acs.chemrestox.0c00528)
Supplement: Supplementary file 1 — tx0c00528_si_001.pdf [file tx0c00528_si_001.pdf]

Supporting Information

Carrier solvents of electronic nicotine delivery systems alter pulmonary surfactant

Nathalie Hayeck[1,2], Carl Zoghzoghi[1], Ebrahim Karam[2,3], Rola Salman[2,3], Nareg Karaoghlanian[2,3], Alan Shihadeh[2,3], Thomas Eissenberg[2,4], Salah Zein El Dine[5] Najat A. Saliba[1,2 *]

[1]Chemistry Department, Faculty of Art and Sciences, American University of Beirut, Beirut Lebanon; [2]Center for the Study of Tobacco Products, Department of Psychology, Virginia Commonwealth University, Richmond, Virginia, USA; [3]Mechanical Engineering Department, Maroun Semaan Faculty of Engineering and Architecture, American University of Beirut, Beirut, Lebanon; [4]Department of Psychology, Virginia Commonwealth University, Richmond Virginia, USA; [5]American University of Beirut, Department of Internal Medicine, Faculty of Medicine, Lebanon

[*]Corresponding author: Najat A. Saliba, Tel: +961 1 350000/3992. E-mail:ns30@aub.edu.lb

ATR-FTIR spectrum of DPPC

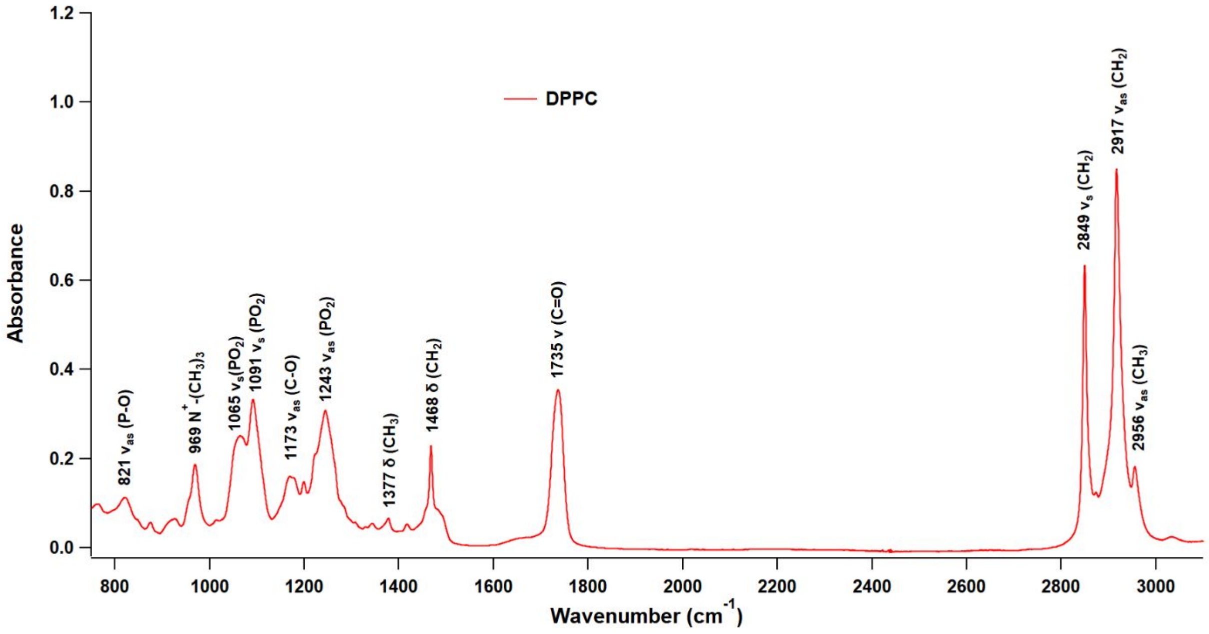

Figure S 1: ATR-FTIR spectrum of DPPC between 750 and 3100 cm^{-1} with an indication of its specific vibrational absorbance.

Effect of PG aerosols on DPPC layer:

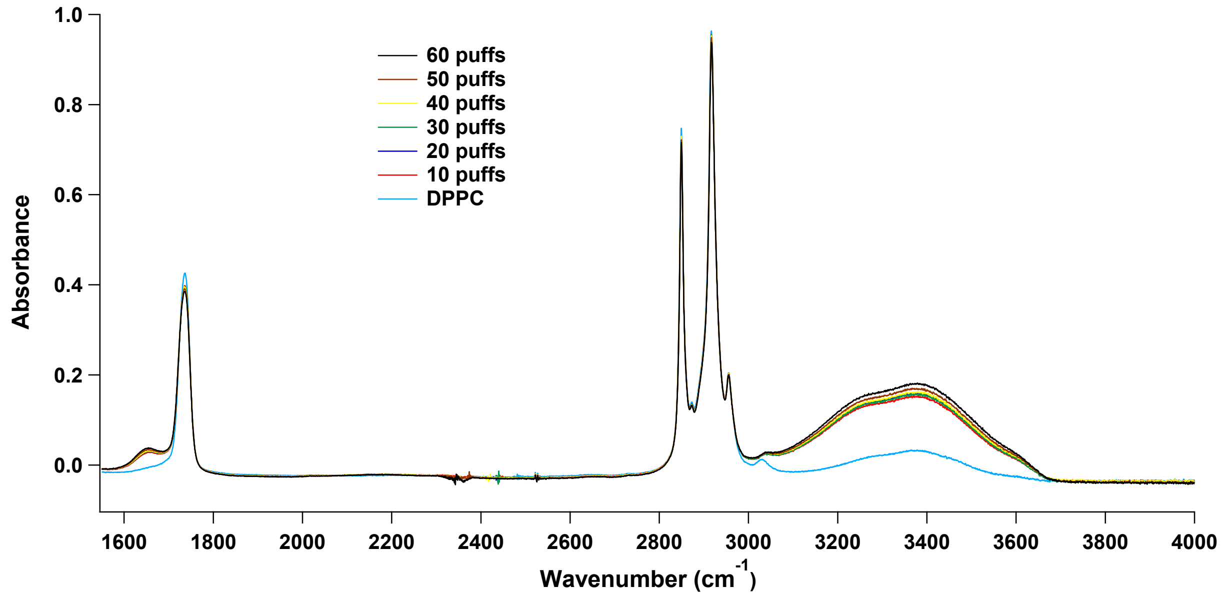

Figure S 2: FTIR spectra of DPPC and after each session of 10 puffs of PG using the ENDS device

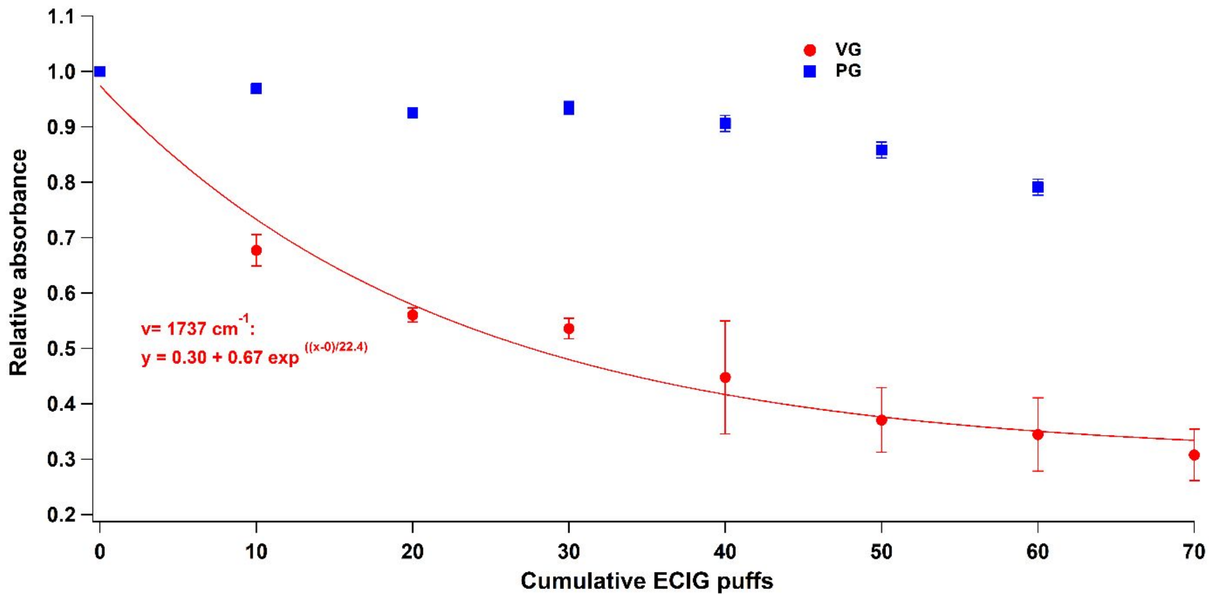

Figure S 3: Relative abundance of one of the affected DPPC vibrational bands (1737 cm^{-1}) as a function of the cumulative number of puffs of VG and PG
